# Postoperative complications and health-related quality of life after oesophageal cancer surgery: a national, population-based cohort study

**DOI:** 10.2340/1651-226X.2024.41290

**Published:** 2024-12-27

**Authors:** Pernilla Lagergren, Asif Johar, Anna Schandl

**Affiliations:** a Surgical Care Science, Department of Molecular Medicine and Surgery, Karolinska Institutet, Karolinska University Hospital, Stockholm, Sweden; b Department of Surgery and Cancer, Imperial College London, London, United Kingdom; c Department of Perioperative- and Intensive Care, Södersjukhuset, Stockholm, Sweden; d Department of Clinical Science and Education, Södersjukhuset, Karolinska Institutet, Stockholm, Sweden

**Keywords:** Oesophageal neoplasm, oesophagectomy, gastro-oesophageal, cancer survivorship

## Abstract

**Background and purpose:**

Surgical resection for oesophageal cancer is technically challenging and associated with a high incidence of complications. This study aimed to assess whether complications influence long-term health-related quality of life (HRQL) in oesophageal cancer survivors.

**Materials and methods:**

This nationwide cohort study included 617 patients who underwent oesophagectomy for cancer in Sweden between 2013 and 2021 with a 1-year follow-up. Complications within 30 days of surgery were categorised into (1) with or without, (2) medical or surgical and (3) according to the Clavien-Dindo classification (0–1, 2–3a, 3b–4). HRQL was assessed with European Organisation for Research and Treatment questionnaires and analysed using multivariable linear regression models, proving mean score differences (MSD) with 95% confidence intervals. The MSDs were adjusted for age, sex, education level, comorbidity, tumour histology, tumour stage, surgical technique and neoadjuvant therapy.

**Results:**

The cohort included 406 patients who survived at least 1 year after oesophageal cancer surgery. Of these, 273 (68%) had at least one complication in the first 30 postoperative days. Long-term HRQL was comparable between patients with or without complications, with or without medical and surgical complications and between different grades of complication severity.

**Interpretation:**

This study suggests that the influence of complications within 30 days of oesophageal cancer surgery on HRQL is minor after 1 year.

## Introduction

To date, surgical removal of the oesophagus remains the mainstay of curative treatment for oesophageal cancer [1]. However, the procedure is technically challenging and is associated with poor prognosis and a high incidence of complications (approx. 50%) [2, 3] with anastomotic leak as the most commonly reported complication followed by postoperative pneumonia [4]. Postoperative complications have been shown to decrease survival in oesophageal cancer patients [5]. There is evidence suggesting that complications can affect health-related quality of life (HRQL). Several studies indicate that severe complications during treatment increase the risk of HRQL deterioration in the short- and long-term [6–9]. In contrast, other studies imply that HRQL is comparable independent of postoperative complications [10, 11]. However, some of the available literature was published before the introduction of minimally invasive procedures and Enhanced Recovery After Surgery (ERAS) programs [12–14] and the overall evidence for the influence of severe complications remains contradictory. Early recognition and treatment of postoperative complications may also vary between countries and healthcare organisations. With more recent data on complications and HRQL, we aimed to investigate whether postoperative complications were associated with reduced HRQL 1 year after oesophageal cancer surgery.

## Methods

### Study design

The study is based on a nationwide prospective cohort entitled the Oesophageal Surgery on Cancer Patients – Adaption and Recovery (OSCAR) study, which includes patients who underwent surgery for oesophageal cancer between 1 January 2013 and 31 March 2021 in Sweden. The study has previously been presented in detail elsewhere [15]. In short, eligible patients were identified through collaboration with pathology departments of the eight operating hospitals in Sweden. The survivors were asked to take part in the study by a project coordinator a year following their operation. Non-Swedish speaking and cognitively impaired patients were excluded. A research nurse visited the homes of all consenting survivors for the collection of patient-reported outcomes. The research nurse guided them through computer-based questionnaires, including open-ended and multiple-choice items. The survivors were followed up regularly for 12 years. For this study, data from the 1-year assessment were used. The study followed the STROBE statement [16].

### Data collection

The retrospective retrieval of patient characteristic data and clinical variables were obtained from medical records and included age, sex at birth, education level, comorbidity, tumour histology, pathological tumour stage, surgical technique, neoadjuvant chemo (radio)therapy and information on complications. A predefined protocol for data collection was used to ensure data consistency. Two researchers reviewed each medical record and an independent person to study cross-validated randomly selected protocols. Histopathology reports provided data on tumour histology, site, and stage. Sociodemographic information was retrieved by linkage to the Swedish Longitudinal integrated database for health insurance and labour market studies (LISA) [17]. Data on comorbidities were retrieved from the Swedish Patient Registry [18] and the Swedish Cancer Registry [19] and were classified according to the Charlson Comorbidity Index score [20, 21]. Information on survival was obtained from The Swedish Register of the Total Population [22].

### Exposure

Postoperative complications, defined as deviations from the normal postoperative course and occurring within 30 days of surgery were considered the exposure. Complications were categorised into (1) occurrence of complications, with or without, (2) pre-specified complication groups (medical- and surgical complications, yes or no) and (3) grades of severity according to the Clavien Dindo system (0–1, 2–3a and 3b–4) [23].

The complications were pre-defined before the data collection began. Medical complications included sepsis (causing clinical symptoms such as fever, chills and the presence of bacteria in the blood); pneumonia (radiologically detected infiltrate with clinical symptoms such as fever, cough or dyspnoea); hepatic insufficiency (progressive jaundice); renal failure (need of dialysis); deep venous thrombosis (radiologically or clinically verified with treatment needs); pulmonary embolism (radiologically detected); myocardial infarction (electrocardiogram or cardiac enzymes verified); atrial fibrillation (newly electrocardiogram detected and treatment required); cerebral infarction/stroke (radiologically verified); and respiratory insufficiency (reintubation or in need of mechanical ventilation).

Surgical complications were postoperative bleeding (> 2,000 mL or requiring reoperation); anastomosis insufficiency (clinically significant or radiologically detected); substitute necrosis (clinically significant ischaemia with ulceration or perforation); thoracic ductus injury (thoracic lymph leakage requiring drainage for more than 7 days or reoperation); intrathoracic abscess or empyema (≥ 3*3 cm radiologically or surgically detected abscess with clinical symptoms such as fever, pain or dyspnoea); intra-abdominal abscess (≥ 3*3 cm radiologically or surgically detected abscess with clinical symptoms such as fever or pain); wound dehiscence (clinically obvious wound rupture); wound infection (causing clinical symptoms and requiring treatment); ileus (radiologically detected ileus in need of surgery); gastric perforation (surgical intervention required); recurrent laryngeal nerve paralysis (laryngeal inspection ascertained); and strictures in anastomosis (endoscopic intervention required).

### Outcome

HRQL was assessed by the European Organization for Research and Treatment of Cancer (EORTC) Quality of Life Questionnaire Core 30 (QLQ-C30) [24] including the modules for gastroesophageal symptoms (QLQ-OG25). The 30-item core questionnaire (QLQ-C30) measures HRQL aspects with five multi-item functional scales (physical, role, cognitive, emotional and social); one global quality of life scale; three symptom scales (fatigue, pain and nausea/vomiting) and six single-items measuring symptoms common among patients with cancer in general (dyspnoea, insomnia, appetite, constipation and diarrhoea) and financial impact [24]. Oesophageal cancer-specific symptoms were measured with the QLQ-OG25 [25], comprising six symptom scales (dysphagia, eating restrictions, reflux, odynophagia, pain and discomfort, and anxiety) and 10 single items (eating in front of others, dry mouth, trouble with taste, body image, trouble swallowing saliva, choking when swallowing, trouble with coughing, trouble talking, weight loss and hair loss) [25]. For the QLQ-C30 and QLQ-OG25, there are four response alternatives: ‘not at all’, ‘a little’, ‘quite a bit’, and ‘very much’. The only exception is the global quality of life scale, which has a seven-graded rating, ranging from 1 (‘very poor’) to 7 (‘excellent’). The questionnaire scores were transformed into 0 to 100 scales where higher scores in the global quality of life score, functional scales and summary score indicate better HRQL, while higher scores in the symptom scales or items correspond to more symptoms. Missing items were managed according to the EORTC scoring manual [26].

### Statistical analysis

Multivariable linear regression models were used to calculate mean score differences (MSD) with 95% confidence intervals (CI), comparing HRQL in patients with postoperative complications with no complications as the reference group. We considered the following eight covariates: age (continuous), sex (male and female), education level (≤ 9, 10–12 or ≥ 13 years of formal education), comorbidity (Charlson comorbidity index score 0, 1, or ≥ 2), tumour histology (adenocarcinoma or squamous cell carcinoma), pathological tumour stage (0–I, II or III–IV), surgical technique (open surgery, minimally invasive and hybrid) and neoadjuvant chemo (radio)therapy (yes or no). To further evaluate whether covariates modified potential associations, stratified analyses were separately performed for the EORTC QLQ-C30 summary score and each of the following variables: age (≤ 59, 60–70 and ≥ 71 years), sex at birth, pathological tumour stage, tumour histology and surgical technique. Because the missing data were low (only 3%), we conducted a complete case analysis. Subgroup analyses were conducted for procedure-specific complications with anastomotic leakage, strictures and gastric tube necrosis as a combined variable, and reoperation within 30 days of main surgery. Since intensive care unit (ICU) stay may serve as an indicator of strain related to complications, differences in HRQL between 0–1 day, 2–3 days, 4–7 days, and ≥8 days of ICU length of stays were explored.

Evidence-based guidelines were used to determine the clinical relevance of medium or large HRQL score differences [27]. If no cut-off level was available, MSDs of 10 to 20 points were regarded as moderate or large clinical differences [28, 29]. To avoid multiple testing, statistical significance was tested at a 5% level of significance only if the MSDs were clinically relevantly moderate or large. All data management and statistical analyses were conducted by a senior biostatistician (AJ) using SAS version 9.4 (Cary, North Carolina).

### Ethical declaration

The study was performed in accordance with the Declaration of Helsinki. Approval was obtained from the Regional Ethical Review Board in Stockholm (2013/844-31/3). All participants provided written informed consent.

## Results

### Patients

In total, 1,013 patients underwent oesophagectomy for cancer during the study period. Among them, 617 were survivors and 406 (66%) completed the questionnaires 1 year after surgery. The main reasons for non-participation were declining consent/not reachable (*n* = 143, 23%), followed by being too sick to participate (*n* = 30, 5%) and 26 (4%) experienced tumour recurrence. Three patients were removed from the analysis because of missing clinical and/or education data. Demographics and clinical variables were balanced between patients with and without complications, except for comorbidities that were more common in patients with complications (*p* < 0.03). The mean age was 66.7 (standard deviation 10.3) and most patients (92%) were men (Table 1). Patients’ characteristics, clinical variables for medical and surgical complications and grades of complication severity classified according to Clavien-Dindo system are presented in Supplementary Tables 1 and 2. In total, 273 (68%) experienced at least one postoperative complication (Table 1). Of the responders, 177 (44%) had medical complications and 138 (34%) had at least one surgical complication, while complications according to Clavien-Dindo system (CD) were distributed as follows: CD 0–1, 146 (36%), CD 2–3a, 152 (38%) and CD 3b–4, 105 (26%). The most common complications were pneumonia (*n* = 77, 19%), followed by anastomotic insufficiency (*n* = 73, 18%) and atrial fibrillation (*n* = 66, 16%) (Table 2). Fifty patients underwent reoperation within 30 days of surgery.

**Table 1:** Patients’ characteristics and clinical variables across patients 1 year after surgery for oesophageal cancer categorised as with or without complications.

Characteristics	All	Complications	
		Without	With
Total number (%)	403 (100)	130 (32)	273 (68)
Age at surgery, mean ± SD	66.7 ± 10.3	65.9 ± 10.4	67.0 ± 10.3
Sex at birth, *n* (%)			
Women	33 (8)	13 (40)	20 (60)
Men	370 (92)	117 (32)	253 (68)
Education level *n* (%)			
< 9 years	99 (25)	32 (32)	67 (68)
10–12 years	190 (47)	63 (33)	127 (67)
≥ 13 years	114 (28)	35 (31)	79 (69)
Charlson Comorbidity Index, *n* (%)			
0	168 (42)	64 (38)	104 (62)
1	138 (34)	44 (32)	94 (68)
≥ 2	97 (24)	22 (23)	75 (77)
Tumour stage, *n* (%)			
0–I	139 (34)	46 (32)	93 (67)
II	127 (32)	46 (36)	81 (64)
III–IV	137 (34)	38 (28)	99 (72)
Tumour histology, *n* (%)			
Adenocarcinoma and high-grade dysplasia	348 (86)	116 (33)	232 (67)
Squamous cell carcinoma	55 (14)	14 (25)	41 (75)
Surgical technique, *n* (%)			
Minimally invasive	150 (37)	48 (32)	102 (68)
Hybrid	138 (34)	46 (33)	92 (67)
Open	115 (29)	36 (31)	79 (69)
Neoadjuvant therapy, *n* (%)			
Yes	323 (80)	108 (33)	215 (67)
No	80 (20)	22 (28)	58 (73)

SD: standard deviation.

**Table 2:** Postoperative complications up to 30 days after surgery for oesophageal cancer presented as numbers (%).

Postoperative complications up to 30 days	
Complications*	Number (%)
No	130 (32)
Medical	177 (44)
Surgical	138 (34)
*Medical complications*	
Sepsis	47 (12)
Pneumonia	77 (19)
Liver insufficiency	1 (0.2)
Renal failure requiring dialysis	1 (0.2)
Deep vein thrombosis	0
Pulmonary embolism	16 (4)
Myocardial infarction	6 (2)
Stroke	0
Respiratory failure	57 (14)
Pulmonary oedema	62 (15)
Atrial fibrillation	66 (16)
*Surgical complications*	
Major bleeding (during surgery)	2 (0.5)
Splenectomy	2
Anastomotic insufficiency	73 (18)
Substitute necrosis	12 (3)
Severe lymph leakage	16 (4)
Gastric perforation	2 (0.5)
Intra-abdominal abscess	10 (2.5)
Intra-thoracic abscess or empyema	31 (8)
Wound infection	29 (7)
Wound rupture	1 (0.2)
Bowel obstruction	5 (1.2)

* Each patient may have had more than one type of complication and may therefore be represented in both groups.

### Postoperative complications and HRQL

The HRQL scales and items for QLQ-C30 and QLQ-OG25 are presented in Tables 3 and 4. The occurrence of postoperative complications (with or without) was not associated with any clinically relevant decrease in global quality of life (adjusted MSD –0.8, 95% CI: –5.7 to 4.0), general functions and symptoms or oesophageal cancer-specific symptoms. Similarly, no associations were seen between the medical or surgical complication categories and deteriorations of any HRQL aspects. Furthermore, no clinically relevant MSD was seen for global quality of life in CD 2–3a (adjusted MSD –0.9, 95% CI: –6.1 to 4.3), or 3b–4 (adjusted MSD –3.2, 95% CI: –9.0 to 2.6), with 0–1 as reference or any other of the HRQL aspects. For procedure-specific complications (anastomotic leakage, strictures and gastric tube necrosis) and reoperation within 30 days, there were no differences in HRQL between groups, except for more dyspnoea for those with complications (adjusted MSD 9.9, 95% CI: 2.6 to 17.1) and (adjusted MSD 11.1, 95% CI: 2.1 to 20.0), respectively (Supplementary Table 3). For ICU length of stay, the only differences in HRQL were found for dyspnoea in patients with ≥ 8 ICU days (adjusted MSD 16.7, 95% CI: 13.6 to 29.9) and anxiety in patients with 4–7 ICU days (adjusted MSD 11.8, 95%CI: 1.4 to 22.2) (Supplementary Table 4). Stratified analyses revealed only few differences in EORTC QLQ-C30 summary score between patients with or without complications in subgroups of age, sex, tumour stage, histology or surgical technique (Table 5).

**Table 3:** Health-related quality of life (HRQL) in patients with and without complications and categorised as medical and surgical complications presented as mean scores (for the referent without complications) and mean score differences (for those with complications) with 95% confidence intervals (CI).

HRQL aspects	All patients		Medical complications		Surgical complications	
	Without complication Mean scores (95%CI)	With complication Mean score differences (95%CI)	Without complications Mean scores (95%CI)	With complication Mean score differences (95%CI)	Without complications Mean scores (95%CI)	With complication Mean score differences (95%CI)
**EORTC QLQ-C30**						
Global quality of life	65.7 (59.8 to 71.5)	–0.8 (–5.7 to 4.0)	65.3 (60.2 to 70.5)	–0.5 (–5.1 to 4.0)	66.6 (61.4 to 71.8)	–3.5 (–8.4 to 1.3)
*Functional scales*						
Physical function	81.5 (76.8 to 86.2)	–2.4 (–6.3 to 1.5)	80.9 (76.7 to 85.0)	–2.4 (–6.0 to 1.3)	81.7 (77.6 to 85.9)	–4.5 (–8.3 to –0.6)
Role function	74.7 (67.3 to 82.1)	2.2 (–3.9 to 8.3)	75.1 (68.6 to 81.6)	2.7 (–3.0 to 8.4)	76.6 (70.0 to 83.1)	–0.7 (–6.8 to 5.4)
Emotional function	79.2 (74.3 to 84.2)	0.5 (–3.6 to 4.5)	80.2 (75.8 to 84.5)	–1.4 (–5.2 to 2.4)	78.8 (74.4 to 83.1)	1.8 (–2.2 to 5.9)
Cognitive function	83.4 (78.3 to 88.4)	–1.4 (–5.5 to 2.8)	83.8 (79.3 to 88.3)	–3.2 (–7.1 to 0.7)	82.8 (78.3 to 87.3)	–0.9 (–5.1 to 3.3)
Social function	79.6 (73.4 to 85.8)	0 (–5.1 to 5.2)	80.4 (74.9 to 85.9)	–1.7 (–6.5 to 3.1)	80.3 (74.7 to 85.8)	–1.5 (–6.6 to 3.7)
*Symptom scales*						
Fatigue	34.8 (28.7 to 40.9)	1.4 (–3.7 to 6.4)	35.4 (30.0 to 40.8)	0.7 (–4.0 to 5.5)	34.5 (29.0 to 39.9)	3.0 (–2.0 to 8.0)
Nausea/vomiting	18.2 (12.9 to 23.5)	0.3 (–4.0 to 4.6)	18.9 (14.3 to 23.6)	–1.1 (–5.2 to 2.9)	17.2 (12.5 to 21.9)	2.9 (–1.4 to 7.2)
Pain	22.3 (16.0 to 28.6)	–1.4 (–6.6 to 3.8)	22.1 (16.5 to 27.6)	–1.8 (–6.7 to 3.1)	21.6 (16.0 to 27.3)	–0.8 (–6.0 to 4.4)
*Symptom items*						
Dyspnoea	27.8 (20.4 to 35.2)	4.2 (–1.9 to 10.4)	30.1 (23.5 to 36.7)	1.6 (–4.2 to 7.3)	27.8 (21.2 to 34.4)	7.0 (0.9 to 13.1)
Insomnia	30.2 (22.1 to 38.2)	–1.5 (–8.1 to 5.1)	29.7 (22.6 to 36.8)	–1.3 (–7.6 to 4.9)	30.3 (23.2 to 37.5)	–2.9 (–9.6 to 3.7)
Appetite loss	22.8 (15.5 to 30.1)	1.2 (–4.8 to 7.2)	23.0 (16.6 to 29.4)	1.4 (–4.2 to 7.1)	21.4 (15.0 to 27.8)	5.3 (–0.7 to 11.3)
Constipation	10.9 (5.8 to 16.1)	–3.2 (–7.4 to 1.0)	9.6 (5.0 to 14.1)	–2.1 (–6.1 to 1.9)	9.5 (4.9 to 14.1)	–2.0 (–6.2 to 2.3)
Diarrhoea	23.9 (16.8 to 31.1)	0 (–5.9 to 5.9)	24.4 (18.1 to 30.7)	–0.9 (–6.5 to 4.6)	24.0 (17.7 to 30.3)	–0.2 (–6.1 to 5.7)
Financial difficulties	7.5 (1.8 to 13.3)	2.1 (–2.5 to 6.8)	8.5 (3.4 to 13.6)	1.2 (–3.2 to 5.6)	7.6 (2.5 to 12.7)	3.3 (–1.4 to 8.0)
**EORTC QLQ-OG-25**						
Body image	17.1 (10.4 to 23.7)	–0.9 (–6.4 to 4.5)	15.6 (9.7 to 21.4)	1.9 (–3.2 to 7.1)	15.8 (9.9 to 21.7)	1.5 (–4.0 to 7.0)
Dysphagia	10.6 (7.0 to 14.3)	–0.7 (–3.7 to 2.3)	10.7 (7.5 to 13.9)	–1.3 (–4.1 to 1.6)	10.4 (7.2 to 13.6)	–0.5 (–3.6 to 2.5)
Eating restrictions	27.7 (22.0 to 33.4)	–0.4 (–5.0 to 4.3)	28.0 (23.0 to 33.0)	–1.3 (–5.7 to 3.1)	27.8 (22.0 to 32.8)	–0.9 (–5.5 to 3.8)
Reflux	28.8 (21.9 to 35.6)	1.2 (–4.5 to 6.8)	28.7 (22.7 to 34.8)	2.0 (–3.3 to 7.4)	30.5 (24.4 to 36.6)	–2.0 (–7.7 to 3.7)
Odynophagia	13.9 (9.1 to 18.8)	1.4 (–2.6 to 5.3)	15.4 (11.1 to 19.6)	–1.1 (–4.9 to 2.7)	14.5 (10.2 to 18.8)	0.9 (–3.1 to 4.9)
Pain and discomfort	20.6 (14.2 to 27.0)	0.6 (–4.7 to 5.9)	22.6 (17.0 to 28.3)	–3.6 (–8.6 to 1.3)	21.0 (15.3 to 26.7)	0.1 (–5.2 to 5.4)
Anxiety	38.4 (31.4 to 45.4)	–2.9 (–8.7 to 2.8)	35.8 (29.6 to 41.9)	–1.3 (–4.1 to 6.7)	37.8 (31.6 to 44.0)	–3.5 (–9.3 to 2.3)
Eating with others	9.4 (4.2 to 14.7)	1.0 (–3.3 to 5.3)	10.1 (5.4 to 14.7)	0.1 (–3.9 to 4.2)	9.4 (4.8 to 14.1)	1.6 (–2.7 to 6.0)
Trouble swallowing saliva	7.1 (2.5 to 11.6)	2.8 (–0.9 to 6.6)	7.6 (3.6 to 11.6)	3.3 (–0.2 to 6.9)	8.7 (4.6 to 12.8)	0.8 (–2.9 to 4.6)
Choking	12.4 (7.1 to 17.7)	2.9 (–1.5 to 7.2)	12.5 (7.8 to 17.2)	4.2 (0.1 to 8.4)	14.4 (9.7 to 19.21)	–0.1 (–4.5 to 4.3)
Dry mouth	34.9 (27.2 to 42.7)	–0.5 (–6.9 to 5.9)	33.7 (26.8 to 40.5)	2.1 (–3.9 to 8.1)	35.0 (28.1 to 41.9)	–0.9 (–7.3 to 5.5)
Coughing	39.5 (32.1 to 46.8)	1.9 (–4.2 to 7.0)	39.6 (33.1 to 46.1)	2.7 (–3.0 to 8.5)	38.4 (31.9 to 44.9)	5.8 (–0.3 to 11.8)
Speech difficulties	5.4 (0.3 to 10.6)	3.3 (–0.9 to 7.6)	7.6 (3.0 to 12.2)	0.5 (–3.6 to 4.5)	5.8 (1.3 to 10.4)	4.6 (0.3 to 8.8)
Taste problems	22.3 (15.5 to 29.2)	–1.0 (–6.6 to 4.7)	22.8 (16.7 to 28.8)	–2.5 (–7.9 to 2.8)	21.6 (15.5 to 27.7)	0.1 (–5.6 to 5.8)
Weight loss	24.1 (15.8 to 32.4)	2.5 (–4.3 to 9.3)	25.3 (18.0 to 32.7)	1.3 (–5.2 to 7.8)	25.7 (18.4 to 33.1)	0.4 (–6.5 to 7.3)

Adjusted for age, sex, education level, comorbidity, pathological tumour stage, tumour histology, surgical technique and neoadjuvant therapy.

EORTC: European Organization for Research and Treatment of Cancer; QLQ-C30: Quality of Life Questionnaire Core 30.

**Table 4:** Health–related quality of life (HRQL) in patients with complications classified according to Clavien-Dindo classification 0–1, 2–3a or 3b–4 presented as mean scores (for the referent Clavien Dindo 0–1) and mean score differences (Clavien Dindi 2–3a and 3b–4) with 95% confidence intervals (CI).

HRQL aspects	Clavien Dindo		
	0–1 Mean scores (95%CI)	2–3a Mean score differences (95%CI)	3b–4 Mean score differences (95%CI)
**EORTC QLQ–C30**			
Global quality of life	66.4 (60.7 to 72.0)	–0.9 (–6.1 to 4.3)	–3.2 (–9.0 to 2.6)
*Functional scales*			
Physical function	82.7 (78.2 to 87.2)	–1.2 (–5.4 to 2.9)	–7.9 (–12.5 to –3.3)
Role function	76.0 (68.9 to 83.2)	1.8 (–4.7 to 8.4)	–1.1 (–8.4 to 6.1)
Emotional function	79.6 (74.9 to 84.4)	0 (–4.4 to 4.4)	–0.2 (–5.0 to 4.7)
Cognitive function	84.0 (79.1 to 88.8)	–0.7 (–5.1 to 3.8)	–4.4 (–9.4 to 0.6)
Social function	79.9 (73.9 to 85.9)	0.3 (–5.3 to 5.8)	–1.1 (–7.2 to 5.1)
*Symptom scales*			
Fatigue	33.7 (28.7 to 39.6)	–0.1 (–5.4 to 5.3)	6.4 (0.5 to 12.4)
Nausea/vomiting	17.7 (12.7 to 22.8)	–1.7 (–6.3 to 3.0)	4.1 (–1.1 to 9.2)
Pain	21.5 (15.4 to 27.6)	–1.3 (–7.0 to 4.3)	0.8 (–5.5 to 7.0)
*Symptom items*			
Dyspnoea	28.0 (20.9 to 35.1)	0.9 (–5.6 to 7.5)	8.0 (0.7 to 15.4)
Insomnia	30.3 (22.5 to 38.0)	–0.5 (–7.7 to 6.7)	–3.2 (–11.2 to 4.7)
Appetite loss	22.3 (15.3 to 29.4)	1.2 (–5.3 to 7.6)	3.0 (–4.2 to 10.2)
Constipation	10.3 (5.3 to 15.2)	–3.9 (–8.5 to 0.7)	–1.0 (–6.1 to 4.1)
Diarrhoea	24.0 (17.1 to 30.9)	2.8 (–3.5 to 9.2)	–3.3 (–10.3 to 3.8)
Financial difficulties	7.3 (1.7 to 12.8)	1.1 (–3.9 to 6.1)	4.5 (–1.1 to 10.1)
**EORTC QLQ–OG–25**			
Body image	16.8 (10.4 to 23.2)	–0.4 (–6.3 to 5.5)	–0.8 (–7.4 to 5.8)
Dysphagia	10.5 (7.0 to 14.3)	–1.1 (–4.4 to 2.1)	0.1 (–3.5 to 3.7)
Eating restrictions	27.7 (22.3 to 33.2)	–0.8 (–5.8 to 4.3)	–0.1 (–5.7 to 5.5)
Reflux	28.9 (22.3 to 35.5)	1.8 (–4.3 to 7.9)	0.3 (–6.5 to 7.1)
Odynophagia	13.3 (8.6 to 17.9)	1.3 (–3.0 to 5.6)	3.8 (–1.0 to 8.6)
Pain and discomfort	21.3 (15.1 to 27.5)	–1.5 (–7.2 to 4.1)	0.9 (–5.4 to 7.2)
Anxiety	37.4 (30.6 to 44.1)	0.1 (–6.1 to 6.3)	–3.3 (–10.2 to 3.6)
Eating with others	10.1 (5.1 to 15.2)	1.0 (–3.6 to 5.7)	–1.1 (–6.3 to 4.1)
Trouble swallowing saliva	7.0 (2.6 to 11.4)	3.8 (–0.2 to 7.8)	2.7 (–1.8 to 7.2)
Choking	11.9 (6.8 to 17.0)	2.1 (–2.6 to 6.8)	5.9 (0.6 to 11.1)
Dry mouth	34.4 (26.9 to 41.8)	–3.5 (–10.3 to 3.4)	4.5 (–3.1 to 12.2)
Coughing	37.8 (30.8 to 44.9)	0.8 (–5.6 to 7.3)	8.8 (1.6 to 16.1)
Speech difficulties	5.7 (0.8 to 10.7)	2.0 (–2.6 to 6.6)	4.4 (–0.7 to 9.5)
Taste problems	22.0 (15.4 to 28.6)	1.0 (–5.0 to 7.1)	–2.1 (–8.9 to 4.7)
Weight loss	24.7 (16.7 to 32.7)	1.2 (–6.2 to 8.6)	2.5 (–5.7 to 10.7)

Adjusted for age, sex, education level, comorbidity, pathological tumour stage, tumour histology, surgical technique and neoadjuvant therapy.

EORTC: European Organization for Research and Treatment of Cancer; QLQ-C30: Quality of Life Questionnaire Core 30.

**Table 5:** Complications (with or without) and EORTC QLQ-C30 summary score one year after surgery for oesophageal cancer (complete case analysis) presented as mean scores (MS) for the referent ‘without complications’ and mean score differences (MSD) for ‘with complications’ with 95% confidence intervals (CI).

Patient and clinical variables	Unadjusted				Adjusted			
	No complications		Complications		No complications		Complications	
	MS (95%CI)	MSD (95%CI)	MS (95%CI)	MSD (95%CI)	MS (95%CI)	MSD (95%CI)	MS (95%CI)	MSD (95%CI)
**All patients**	80.6 (78.2 to 83.1)	Reference	80.0 (78.3 to 81.7)	–0.6 (–3.6 to 2.4)	77.3 (74.3 to 80.4)	Reference	77.5 (73.9 to 81.1)	–0.2 (–3.2 to 2.8)
**Age**								
< 60 years	79.2 (74.2 to 84.3)	Reference	71.7 (67.6 to 75.7)	–7.6* (–14.0 to –1.1)	75.0 (73.3 to 82.5)	Reference	70.0 (64.1 to 73.8)	–6.0 (–12.7 to 0.6)
60–70 years	81.1 (77.3 to 84.8)	Reference	80.3 (77.7 to 82.9)	–0.7 (–5.3 to 3.8)	77.9 (73.3 to 82.5)	Reference	77.8 (74.0 to 81.6)	–0.1 (–4.7 to 4.5)
> 70 years	81.0 (77.0 to 85.0)	Reference	83.2 (80.6 to 85.7)	2.2 (–2.6 to 6.9)	78.7 (71.0 to 83.5)	Reference	80.9 (77.4 to 84.5)	2.2 (–2.6 to 7.0)
**Sex at birth**								
Men	80.6 (78.0 to 83.2)	Reference	80.4 (78.6 to 82.2)	–0.2 (–3.3 to 3.0)	77.4 (74.0 to 80.9)	Reference	77.8 (75.2 to 80.5)	0.4 (–2.7 to 3.5)
Women	85.1 73.4 to 88.9)	Reference	75.3 (69.0 to 81.5)	–5.8 (–15.8 to 4.1)	80.7 (72.8 to 88.6)	Reference	74.8 (68.5 to 81.0)	–6.0 (–15.9 to 3.9)
**Tumour stage**								
0–I	82.4 (78.2 to 86.6)	Reference	81.8 (78.9 to 84.7)	–0.6 (–5.7 to 4.5)	79.3 (74.3 to 84.3)	Reference	79.5 (75.5 to 83.4)	0.2 (–4.9 to 5.3)
II	80.1 (75.9 to 84.2)	Reference	78.5 (75.4 to 81.7)	–1.5 (–6.7 to 3.7)	77.1 (72.2 to 82.1)	Reference	75.6 (71.7 to 79.6)	–1.5 (–6.6 to 3.7)
III–IV	79.2 (74.6 to 83.7)	Reference	79.6 (76.7 to 82.4)	0.4 (–5.0 to 5.8)	76.1 (70.9 to 81.2)	Reference	76.9 (70.9 to 81.2)	0.8 (–4.5 to 6.1)
**Histology**								
Adenocarcinoma	80.6 (78.0 to 83.2)	Reference	80.7 (78.9 to 82.6)	0.1 (–3.1 to 3.3)	78.9 (75.3 to 82.6)	Reference	79.3 (76.2 to 82.5)	0.4 (–2.8 to 3.6)
Squamous cell carcinoma	80.6 (73.1 to 82.6)	Reference	79.3 (76.2 to 82.5)	–4.4 (–13.0 to 4.3)	78.7 (71.0 to 86.3)	Reference	74.3 (69.6 to 79.1)	–4.3 (–13.0 to 4.3)
**Surgery technique**								
Minimally invasive	80.4 (76.4 to 84.5)	Reference	79.2 (76.4 to 82.0)	–1.2 (–6.2 to 3.8)	78.1 (73.2 to 83.1)	Reference	77.0 (73.2 to 80.8)	–1.1 (–6.0 to 3.8)
Hybrid	82.0 (77.9 to 86.2)	Reference	81.6 (78.8 to 84.6)	–0.4 (–5.5 to 4.7)	78.5 (73.6 to 83.4)	Reference	78.1 (74.0 to 82.2)	–0.4 (–5.4 to 4.6)
Open	79.1 (74.4 to 83.7)	Reference	79.2 (76.0 to 82.3)	0.1 (–5.5 to 5.8)	75.7 (70.4 to 80.9)	Reference	77.0 (73.1 to 80.8)	1.3 (–4.3 to 6.9)

Adjusted for age, sex, education level, comorbidity, pathological tumour stage, tumour histology, surgical technique and neoadjuvant therapy.

* Clinically relevantly moderate differences (*p* < 0.05).

EORTC: European Organization for Research and Treatment of Cancer; QLQ-C30: Quality of Life Questionnaire Core 30.

## Discussion

This study suggests that postoperative complications occurring within 30 days of oesophageal cancer resection are not associated with deteriorated HRQL at 1-year follow-up but are comparable between groups with and without complications.

The study has some strengths and weaknesses. The prospective population-based design counteracts selection bias. Moreover, the large sample, complete follow-up, and high response rate of the questionnaires ensure statistical power and clinically meaningful conclusions. However, the following limitations must be acknowledged: The individual pre-HRQL remains unknown. The study only included patients who survived at least 1 year after surgery and those who responded to the questionnaires. This may have induced a risk of selection bias towards excluding patients with poorer HRQL especially since one of the main reasons for declining participation was recurrence or being too sick to participate. The study included all surgically treated oesophageal cancer survivors in Sweden who had undergone recent treatments including neoadjuvant therapy and the results should therefore be relatively generalisable to healthcare settings with similar organisations and treatment routines.

A systematic review and meta-analysis of 10 studies including 2,181 patients with oesophageal cancer showed that patients experiencing severe postoperative complications lived for 8.6 (CI: –12.5 to –4.7) months less on average compared to those with no complications [30]. Therefore, it seems likely to assume that severe postoperative complications also affect HRQL. Though much research includes complications as a confounding variable in their analysis, few observational studies look into the impact on HRQL, and the available studies have conflicting results. Studies conducted in early year 2000 indicated that severe postoperative complications had a major impact on HRQL. A Swedish population-based cohort study of 153 oesophageal cancer 5-year survivors found that severe postoperative complications were associated with HRQL deteriorations, specifically dyspnea (MSD 15, 95%CI: 6 to 23), fatigue (MSD 13, 95%CI: 5 to 20) and eating restrictions (MSD 10, 95%CI: 2 to 17) [8]. These long-term reductions in HRQL seem to persist up to 15 years after oesophagectomy [6, 31, 32]. However, all included patients in that study cohort underwent open surgical resection. Even though a systematic review of 15 original research articles showed limited HRQL benefits in favour of minimally invasive surgery in comparison with open surgery [33], other recent studies have shown more positive findings for minimally invasive surgery. A European cross-sectional study of 645 individuals diagnosed with oesophageal cancer from 2010 to 2016 found comparable HRQL between patients with and without postoperative complications [10]. A European randomised controlled trial conducted between 2009 and 2011 compared HRQL between 64 1-year survivors of minimally invasive and open surgery, and found that HRQL, especially physical activity, global health and pain, was favourable for those who underwent minimally invasive oesophagectomy [34]. In a retrospective Chinese cohort study of 140 patients who underwent minimally invasive surgery and open surgery with cervical anastomosis between 2013 and 2014, the occurrence of postoperative complications and HRQL ratings were similar between groups [35].

Combining the findings of this study with the previous literature makes it reasonable to argue that the influence of complications within 30 days of surgery on HRQL is minor for patients who undergo surgery today. Most complications may be short-term of character, which may explain the lack of impact on HRQL 1 year after surgery. Moreover, during the last decade, earlier tumour detection, careful patient selection for surgery, and appropriate treatment of postoperative complications may have contributed to improved patient outcomes. Still, it should be recalled that compared to a background population, there is a high symptom burden and relatively low HRQL among oesophageal cancer survivors, independent of complications (or not) [36]. This study provides a better understanding of the long-term effects of postoperative complications in this patient group. The results can be useful in organising postoperative healthcare so signs of complications can be prevented or detected at an early stage. Surgery and surveillance of complications seem to have improved during the last decade, but HRQL among this patient group remains impaired. This finding indicates a need for long-term follow-up of HRQL and effective symptom treatment programs, which hopefully will improve the survivors’ health and well-being.

## Conclusion

In conclusion, the findings from this nationwide cohort study with measures taken to avoid selection bias and confounding, indicate that postoperative complications have a minor impact on HRQL in surgically treated oesophageal cancer patients 1 year after treatment. However, the negative impact of complications on HRQL may be stronger closer to the surgery or may come as negative late effects in the later phase of the follow-up and should therefore be further studied.

## Supplementary Material

Postoperative complications and health-related quality of life after oesophageal cancer surgery: a national, population-based cohort study

## Data Availability

The data that support the findings of this study are available upon request by the corresponding author [AS].
