# Supplementary material for: Postoperative complications and health-related quality of life after oesophageal cancer surgery: a national, population-based cohort study
Source: Acta Oncol. 2024 Dec 27;63:41290. doi: 10.2340/1651-226X.2024.41290 (PMC11697142; doi:10.2340/1651-226X.2024.41290)
Supplement: Postoperative complications and health-related quality of life after oesophageal cancer surgery: a national, population-based cohort study [file AO-63-41290-s1.pdf]

Supplementary material has been published as submitted. It has not been copyedited, or typeset by Acta Oncologica

Supplementary Table 1. Patient characteristics and clinical variables across patients 1 year after surgery for oesophageal cancer classified as medical and surgical complications.

	Complications			
Characteristics	Medical		Surgical	
	Without	With	Without	With
Total number(%)	226 (56)	177 (44)	265 (66)	138 (34)
Age at surgery, mean±SD	66.3±11.1	67.2±9.3	66.5±11.3	67.1±8.1
Sex at birth, n(%)				
Women	21 (64)	12 (36)	22 (69)	10 (31)
Men	205 (55)	165 (45)	243 (66)	127 (34)
Education level, n (%)				
<9 years	52 (53)	47 (47)	68 (69)	31 (31)
10-12 years	111 (58)	79 (42)	120 (63)	70 (37)
≥13 years	63 (55)	51 (45)	77 (68)	37 (32)
Charlson Comorbidity Index, n (%)				
0	100 (60)	68 (40)	129 (77)	39 (23)
1	82 (59)	56 (41)	79 (57)	59 (43)
≥2	44 (45)	53 (55)	57 (59)	40 (41)
Tumour stage, n (%)				
0-I	76 (55)	63 (45)	92 (66)	47 (34)
II	77 (61)	50 (39)	83 (65)	44 (35)
III-IV	73 (53)	64 (47)	90 (66)	47 (34)
Tumour histology, n (%)				
Adenocarcinoma and high-grade dysplasia	195 (56)	153 (44)	237 (68)	111 (32)
Squamous cell carcinoma	31 (56)	24 (46)	28 (51)	27 (49)
Surgical technique, n (%)				
Minimally invasive	85 (57)	65 (43)	96 (64)	54 (36)
Hybrid	85 (62)	53 (38)	94 (68)	44 (32)
Open	56 (49)	59 (51)	75 (65)	40 (35)
Neoadjuvant therapy, n (%)				
Yes	216 (67)	107 (33)	186 (58)	137 (42)
No	49 (61)	31 (39)	40 (50)	40 (50)

SD=standard deviation

Supplementary Table 2. Patient characteristics and clinical variables across patients 1 year after surgery for oesophageal cancer classified according to Clavien Dindo 0-1, 2-3a and 3b-4.

	Clavien Dindo classification		
Characteristics	0-1	2 – 3a	3b - 4
Total number (%)	146 (36)	152 (38)	105 (26)
Age at surgery, mean±SD	66.5±10.1	66.8±11.7	66.7±8.4
Sex at birth, n(%)			
Women	15 (45)	9 (27)	9 (27)
Men	131 (35)	143 (39)	96 (26)
Education level n (%)			
<9 years	36 (36)	37 (37)	26 (26)
10-12 years	71 (37)	67 (35)	52 (27)
≥13 years	39 (34)	48 (42)	27 (24)
Charlson Comorbidity Index, n (%)			
0	68 (40)	65 (39)	35 (21)
1	49 (36)	46 (33)	43 (31)
≥2	29 (30)	41 (42)	27 (28)
Tumour stage, n (%)			
0-I	52 (37)	51 (37)	36 (26)
II	48 (38)	45 (35)	34 (27)
III-IV	46 (34)	56 (41)	35 (26)
Tumour histology, n (%)			
Adenocarcinoma and high-grade dysplasia	130 (37)	133 (38)	85 (24)
Squamous cell carcinoma	16 (29)	19 (34)	20 (36)
Surgical technique, n (%)			
Minimally invasive	53 (35)	60 (40)	37 (25)
Hybrid	53 (38)	54 (39)	31 (22)
Open	40 (35)	38 (33)	37 (32)
Neoadjuvant therapy, n (%)			
Yes	119 (37)	120 (37)	84 (26)
No	27 (34)	32 (40)	21 (26)

SD=standard deviation

Supplementary table 3. Health-related quality of life (HRQL) in patients with and without a) combined anastomotic leakage, strictures and gastric tube necrosis, b) reoperations presented as mean scores (for the referent without complications) and mean score differences (for those with complications) with 95% confidence intervals (CI).

**HRQL aspects**	**Anastomotic leakage, strictures and gastric tube necrosis**		**Reoperation within 30 days**	
	Without (n=337)	With (n=79)	Without (n=361)	With (n=50)
	Mean Scores (95%CI)	Mean Score Differences (95%CI)	Mean Scores (95%CI)	Mean Score Differences (95%CI)
**EORTC QLQ-C30**				
Global quality of life	66.0 (61.0 to 71.0)	-3.4 (-9.2 to 2.3)	65.4 (60.5 to 70.3)	-6.3 (-13.3 to 0.8)
Functional scales				
Physical function	81.8 (77.9 to 85.8)	-7.6 (-12.1 to 3.0)	80.6 (76.7 to 84.5)	-9.2 (-14.8 to -3.6)
Role function	77.6 (71.2 to 83.9)	-4.8 (-12.0 to 2.5)	76.8 (70.7 to 82.9)	-10.1 (-18.9 to -1.3)
Emotional function	80.0 (75.8 to 84.2)	-1.6 (-6.4 to 3.3)	79.8 (75.8 to 83.9)	-0.7 (-6.5 to 5.1)
Cognitive function	83.4 (79.0 to 87.7)	-3.7 (-8.7 to 1.3)	82.2 (78.0 to 86.5)	-1.6 (-7.7 to 4.5)
Social function	80.8 (75.5 to 86.1)	-4.2 (-10.4 to 1.8)	80.0 (74.3 to 84.8)	-3.7 (-11.2 to 3.8)
Symptom scales				
Fatigue	33.8 (28.7 to 39.0)	7.0 (1.0 to 12.9)	35.4 (30.3 to 40.5)	6.7 (-0.7 to 14.0)
Nausea/vomiting	17.2 (12.7 to 21.7)	4.4 (-0.7 to 9.6)	18.5 (14.0 to 22.9)	0.2 (-6.1 to 6.6)
Pain	20.4 (15.0 to 25.8)	3.3 (-2.9 to 9.5)	21.1 (15.8 to 26.4)	3.2 (-4.5 to 10.8)
Symptom items				
Dyspnoea	28.1 (21.8 to 34.5)	9.9 (2.6 to 17.1)*	29.9 (23.6 to 36.1)	11.1 (2.1 to 20.0)*
Insomnia	29.5 (22.6 to 36.4)	-1.7 (-9.6 to 6.3)	29.0 (22.2 to 36.7)	-6.0 (-15.7 to 3.4)
Appetite loss	22.0 (15.8 to 28.2)	6.3 (-0.9 to 13.4)	22.0 (15.9 to 28.0)	9.0 (0.2 to 17.8)
Constipation	8.2 (3.8 to 12.6)	1.8 (-3.3 to 6.8)	8.6 (4.2 to 12.9)	2.8 (-3.5 to 9.0)
Diarrhoea	23.2 (17.1 to 29.3)	2.6 (-4.4 to 9.6)	24.8 (18.8 to 30.8)	-5.1 (-13.8 to 3.5)
Financial difficulties	7.6 (2.7 to 12.5)	5.3 (-0.3 to 10.9)	9.0 (4.2 to 13.9)	3.0 (-3.9 to 9.9)
**EORTC QLQ-OG-25**				

Body image	15.6 (9.9 to 21.2)	3.2 (-3.4 to 9.7)	17.3 (11.8 to 22.9)	-4.0 (-12.0 to 4.0)
Dysphagia	10.0 (6.9 to 13.1)	0.5 (-3.1 to 4.1)	10.2 (7.2 to 13.3)	1.7 (-2.8 to 6.1)
Eating restrictions	27.1 (22.2 to 31.9)	1.4 (-4.1 to 7.0)	27.8 (23.1 to 32.6)	0.4 (-6.4 to 7.3)
Reflux	30.8 (25.0 to 36.7)	-4.7 (-11.4 to 2.1)	30.6 (24.9 to 36.4)	-3.8 (-12.2 to 4.5)
Odynophagia	14.0 (9.9 to 18.2)	3.2 (-1.6 to 8.0)	14.9 (10.9 to 19.0)	-1.2 (-7.1 to 4.7)
Pain and discomfort	20.9 (15.4 to 26.4)	0.6 (-5.7 to 6.9)	20.9 (15.5 to 26.2)	-1.2 (-8.9 to 6.5)
Anxiety	36.8 (30.8 to 42.7)	-1.5 (-8.4 to 5.3)	37.3 (31.5 to 43.1)	-1.4 (-9.8 to 7.0)
Eating with others	10.1 (5.6 to 14.6)	0.1 (-5.1 to 5.3)	10.4 (6.0 to 14.8)	0.4 (-6.0 to 6.8)
Trouble swallowing saliva	8.6 (4.7 to 12.5)	1.8 (-2.7 to 6.3)	9.0 (5.2 to 12.9)	2.5 (-3.0 to 8.0)
Choking	14.0 (9.4 to 18.5)	1.5 (-3.7 to 6.8)	14.1 (9.7 to 18.6)	3.2 (-3.2 to 9.6)
Dry mouth	33.1 (26.5 to 39.8)	5.5 (-2.1 to 13.1)	33.4 (26.9 to 39.9)	7.4 (-2.0 to 16.7)
Coughing	38.6 (32.4 to 44.8)	8.4 (1.2 to 15.7)	40.5 (34.3 to 46.6)	6.5 (-2.5 to 15.6)
Speech difficulties	7.3 (2.9 to 11.8)	1.7 (-3.4 to 6.9)	7.3 (3.0 to 11.6)	6.0 (-0.2 to 12.3)
Taste problems	22.5 (16.6 to 28.3)	-3.0 (-9.8 to 3.7)	22.0 (16.2 to 27.7)	1.8 (-6.5 to 10.1)
Weight loss	25.1 (18.0 to 32.2)	3.0 (-5.2 to 11.3)	27.3 (20.3 to 34.2)	-6.8 (-17.0 to 3.2)

Adjusted for age, sex, education level, comorbidity, pathological tumour stage, tumour histology, surgical technique and neoadjuvant therapy. *Clinically relevantly medium differences ($p<0.05$)

Supplementary table 4. Health-related quality of life (HRQL) in patients and duration of ICU length of stay, categorized as 2-3 days, 4-7 days and ≥8 days with 0-1 day as the referent, presented as mean and mean score differences with 95% confidence intervals (CI).

**HRQL aspects**	**Length of Intensive Care Unit stay**			
	0-1 day (referent) n=107	2-3 days n=72	4-7 days n=38	≥8 days n=26
	Mean Scores (95%CI)	Mean Score Differences (95%CI)	Mean Score Differences (95%CI)	Mean Score Differences (95%CI)
**EORTC QLQ-C30**				
Global quality of life	63.1 (57.0 to 69.3)	-2.6 (-9.6 to 4.5)	-1.6 (-10.2 to 7.0)	2.2 (-7.9 to 12.3)
Functional scales				
Physical function	80.6 (75.7 to 85.4)	-4.8 (-10.4 to 0.7)	-3.3 (-10.1 to 3.5)	-8.0 (-16.1 to 0)
Role function	77.8 (70.0 to 85.5)	-8.3 (-17.2 to 0.5)	-2.8 (-13.6 to 8.1)	-6.6 (-19.3 to 6.3)
Emotional function	79.3 (74.0 to 84.6)	-0.1 (-6.2 to 5.9)	-7.4 (-14.8 to 0)	-2.0 (-10.7 to 6.7)
Cognitive function	83.3 (78.0 to 88.5)	-4.0 (-9.9 to 2.0)	-5.8 (-13.1 to 1.5)	-3.0 (-11.6 to 5.6)
Social function	78.8 (72.0 to 85.6)	-4.7 (-12.5 to 3.1)	2.5 (-7.0 to 12.1)	-1.7 (-12.9 to 9.6)
Symptom scales				
Fatigue	36.7 (30.4 to 43.0)	1.9 (-5.3 to 9.1)	3.6 (-5.2 to 12.4)	10.8 (0.4 to 21.2)
Nausea/vomiting	19.3 (13.9 to 24.8)	3.3 (-2.9 to 9.5)	-0.8 (-8.5 to 6.8)	3.5 (-5.5 to 12.5)
Pain	23.0 (16.5 to 29.5)	3.2 (-4.2 to 10.6)	3.8 (-5.3 to 12.9)	-7.5 (-18.3 to 3.2)
Symptom items				
Dyspnoea	29.7 (21.7 to 37.6)	0.2 (-8.9 to 9.3)	10.1 (-1.1 to 21.3)	16.7 (3.6 to 29.9)*
Insomnia	29.0 (20.1 to 37.9)	4.8 (-5.4 to 15.0)	-2.0 (-14.5 to 10.5)	-3.9 (-18.6 to 10.8)
Appetite loss	23.1 (15.0 to 31.1)	2.3 (-6.9 to 11.4)	1.6 (-9.7 to 12.8)	11.0 (-2.4 to 24.5)
Constipation	10.1 (4.8 to 15.4)	-3.4 (-9.5 to 2.7)	1.9 (-5.6 to 9.4)	-4.0 (-12.7 to 4.8)
Diarrhoea	24.4 (17.0 to 31.9)	2.6 (-5.9 to 11.1)	0.5 (-10.0 to 10.9)	0.1 (-12.2 to 12.4)
Financial difficulties	9.8 (2.9 to 16.7)	3.6 (-4.2 to 11.5)	1.4 (-8.2 to 11.0)	3.7 (-7.9 to 15.2)
**EORTC QLQ-OG-25**				
Body image	14.5 (7.4 to 21.7)	7.0 (-1.2 to 15.1)	5.8 (-4.3 to 15.8)	7.7 (-4.2 to 19.5)
Dysphagia	13.0 (9.1 to 16.9)	-1.3 (-5.7 to 3.1)	-4.1 (-9.4 to 1.4)	-1.7 (-8.2 to 4.7)

Eating restrictions	28.3 (22.4 to 34.2)	0.6 (-6.1 to 7.2)	-1.2 (-9.4 to 7.0)	3.9 (-6.0 to 13.7)
Reflux	33.0 (25.2 to 40.7)	1.5 (-7.4 to 10.3)	1.6 (-9.2 to 12.4)	0.9 (-12.1 to 13.9)
Odynophagia	13.4 (8.5 to 18.2)	7.0 (1.4 to 12.5)	7.6 (0.8 to 14.4)	2.8 (-5.2 to 10.8)
Pain and discomfort	24.0 (17.1 to 31.0)	2.1 (-5.8 to 10.0)	3.9 (-5.8 to 13.7)	-4.7 (-16.1 to 6.8)
Anxiety	36.6 (29.1 to 44.0)	1.2 (-7.3 to 9.7)	11.8 (1.4 to 22.2)*	-7.4 (-19.7 to 4.9)
Eating with others	11.1 (5.2 to 16.9)	-1.6 (-8.3 to 5.0)	-8.0 (-16.1 to 0.2)	0 (-9.6 to 9.6)
Trouble swallowing saliva	8.5 (3.1 to 14.0)	3.3 (-2.9 to 9.5)	-0.7 (-8.4 to 6.8)	7.2 (-1.8 to 16.1)
Choking	17.0 (11.2 to 22.8)	4.3 (-2.4 to 10.9)	-4.8 (-12.9 to 3.3)	6.0 (-3.6 to 15.6)
Dry mouth	37.6 (28.9 to 46.3)	-2.6 (-12.6 to 7.3)	-1.7 (-13.9 to 10.5)	3.3 (-11.1 to 17.7)
Coughing	40.0 (32.2 to 48.0)	3.2 (-5.9 to 12.3)	1.8 (-9.4 to 12.9)	9.3 (-3.8 to 22.4)
Speech difficulties	8.5 (2.6 to 14.4)	3.3 (-3.5 to 10.1)	2.3 (-5.9 to 10.6)	0.6 (-9.1 to 10.3)
Taste problems	23.3 (15.6 to 30.9)	0.9 (-7.9 to 9.6)	0.3 (-10.4 to 11.0)	-8.8 (-21.5 to 3.8)
Weight loss	25.1 (16.0 to 34.1)	3.6 (-6.7 to 13.9)	10.6 (-2.0 to 12.2)	2.1 (-12.7 to 17.0)

Adjusted for age, sex, education level, comorbidity, pathological tumour stage, tumour histology, surgical technique and neoadjuvant therapy. *Clinically relevantly medium or large differences (p<0.05)
